# Nut and seed consumption is inversely associated with metabolic syndrome in females but not males: findings from the 2005–2018 NHANES data

**DOI:** 10.1007/s00394-023-03157-1

**Published:** 2023-04-28

**Authors:** Tommy H. T. Wong, Elena S. George, Gavin Abbott, Robin M. Daly, Ekavi N. Georgousopoulou, Sze-Yen Tan

**Affiliations:** 1 School of Public Health, Li Ka Shing, Faculty of Medicine, The University of Hong Kong, Pokfulam, Hong Kong; 2 Institute for Physical Activity and Nutrition (IPAN), School of Exercise and Nutrition Sciences, Deakin University, Geelong, VIC 3220 Australia; 3 Discipline of Nutrition and Dietetics, University of Canberra, Canberra, Australia

**Keywords:** Nuts, Seeds, Metabolic syndrome, Adult, Glucose, Triglycerides, Central obesity, Blood pressure, HDL-cholesterol

## Abstract

**Purpose:**

To assess the association between nut and seed consumption, both combined and separately, and metabolic syndrome and its components, including fasting glucose, triglycerides, high-density lipoprotein (HDL) cholesterol, central obesity, and blood pressure.

**Methods:**

This cross-sectional analysis used data from 22,687 adults (aged ≥ 18 years) involved in seven cycles (2005–2018) of the National Health and Nutrition Examination Survey (NHANES). Habitual nut and seed intakes were estimated by the Multiple Source Method using data from two 24-h dietary recalls. Metabolic syndrome was ascertained using biochemical data and self-reported medication use. Sex-specific effect estimates were obtained using logistic and linear regressions adjusting for lifestyle and socioeconomic confounders.

**Results:**

Compared to non-consumers, female, but not male, habitual consumers of either nuts or seeds had lower odds of having metabolic syndrome (OR: 0.83, 95% CI 0.71, 0.97). Both nut intake alone and seed intake alone were inversely associated with high fasting glucose and low HDL-cholesterol in females compared to non-consumers. When restricted to habitual consumers only, the combined intake of nuts and seeds at 6 g/day was associated with the lowest triglycerides and highest HDL-cholesterol in females. Combined consumption of nuts and seeds up to one ounce-equivalent (15 g) per day, but not in higher intake levels, was inversely associated with metabolic syndrome, high fasting glucose, central obesity, and low HDL-cholesterol in females.

**Conclusions:**

Nut and seed consumption, both separately or combined, below 15 g/day was inversely associated with metabolic syndrome and its component conditions in females but not males.

**Supplementary Information:**

The online version contains supplementary material available at 10.1007/s00394-023-03157-1.

## Introduction

Metabolic syndrome is a condition that involves the co-occurrence of multiple metabolic abnormalities, including high fasting glucose, dyslipidemia, high blood pressure, and/or central obesity [1]. It is estimated that 20 to 30% of the global population has metabolic syndrome [2–5] and individuals with this condition have a higher risk of developing type 2 diabetes [6], cardiovascular diseases [7], and a higher mortality rate [8]. Although dietary-based interventions are considered an important strategy for the management of metabolic syndrome [9], many questions remain as to which modifiable dietary factors may play a role to reduce the risk of or manage metabolic syndrome.

Nuts and seeds are considered an important component of a healthy diet in dietary guidelines in several countries [10–12] and this is attributed to their plant-based origin while being a source of high quality protein, as well as containing high amounts of mono- and poly-unsaturated fats, dietary fiber, micronutrients, and phytochemicals with antioxidative properties [13]. Habitual nut consumption has been associated with improved weight management [14] and a lower risk of cardiovascular and all-cause mortality [15, 16], but the association with the risk of metabolic syndrome has been inconsistent [17, 18]. Furthermore, few studies have investigated the health benefits of seeds alone even though they have comparable nutrient profiles to nuts and belong to the same food group within dietary guidelines. Due to the similarities in nutritional profiles, nuts and seeds are hypothesized to provide similar health benefits [19]. However, current evidence surrounding the metabolic health benefits of seeds alone is limited to randomized controlled trials [20–23]. For instance, flaxseed intake decreased blood pressure and sesame seeds intake lowered fasting blood glucose [20, 23]. However, results regarding the effect of flaxseed intake on fasting blood glucose were inconsistent [21], and no significant changes in metabolic syndrome markers were observed with chia seeds consumption [22]. Nonetheless, high heterogeneity in the characteristics of participants and the results was noted in all systematic reviews [20–23], thus the quality of evidence was generally considered low or very low based on the Grading of Recommendations Assessment, Development, and Evaluation (GRADE) approach. More importantly, the association between the intake of seeds, either alone or together with nuts as a food group, and metabolic syndrome has not been assessed before. Therefore, the aim of this study was two-fold: (1) to assess the difference in the odds of metabolic syndrome and its related components [high fasting glucose, high triglycerides, central obesity, high blood pressure, and low high-density lipoprotein cholesterol (HDL-cholesterol)] between consumers and non-consumers of nuts and/or seeds, and (2) to examine the presence of a dose–response association between nut and/or seed consumption and metabolic syndrome.

## Methods

### Study population

This study used data collected from males and females aged 18 years and over that participated in the National Health and Nutrition Examination Survey (NHANES), which used a multistage probability sampling procedure to obtain nationally representative estimates of the health and nutritional status of noninstitutionalized residents in the United States [24]. In each survey, participants provided information regarding demographics, socioeconomic status, diet, and health in an interview, underwent medical and physiological examinations, and provided fasted blood samples in which various biomarkers were measured. In this analysis, we included participants aged 18 years or older from seven cycles (2005–2006, 2007–2008, 2009–2010, 2011–2012, 2013–2014, 2015–2016, 2017–2018). The survey protocols for NHANES 2005–2018 (Protocol #2005–06, Protocol #2011–17, and Protocol #2018–01) were approved by the National Centre for Health Statistics (NCHS) Research Ethics Review Board, and all participants provided their informed consent. Further inclusion criteria were that participants had to have completed two reliable 24-h recalls, and have complete data on relevant covariates (age, sex, ethnicity, ratio of family income to poverty, smoking status, alcohol intake, Healthy Eating Index 2015 (HEI 2015), physical activity *z*-score, and prior cardiovascular event).

### Nut and seed intake

Nut and seed intake data were obtained using two 24-h dietary recalls that were carried out on non-consecutive days using the Automated Multiple Pass Method (AMPM), the details of which were previously published [25]. In this analysis, nuts and seeds included the following: (1) nuts: almonds, almond butter, Brazil nuts, cashews, cashew butter, hazelnuts, macadamias, pecans, pine nuts, pistachios, walnuts, peanuts, and peanut butter, and (2) seeds: pumpkin seeds, flaxseeds, sesame seeds, sesame butter, sunflower seeds, psyllium seeds, and chia seeds [26]. In addition to those eaten alone, nuts and seeds that were a component of a meal or an ingredient of food products were also included by searching the Food Commodity Intake Database (FCID), containing recipes for composite food products [27]. Only 24-h recalls that were reliable and met the minimum criteria established by NHANES were used in this study [24]. The usual nut and seed intake were then estimated using the Multiple Source Method (MSM) [28], which is a statistical method for estimating usual dietary intake for episodically consumed foods in individuals and was validated in a large epidemiological study [29]. We included age, sex, product of age and sex, and days of recall (weekday/weekend) as covariates when estimating usual nut and seed intake in this study. Furthermore, we categorized the combined intake of nuts and seeds using cut-offs informed by our previous study: non-consumers (0 g/day), 0.1–15.0 g/day, 15.1–29.9 g/day, and 30.0 g/day or above [26]. Based on the U.S dietary guidelines 2020 [10], an ounce-equivalent of nuts and seeds is defined as ½ oz, which translates to approximately 15 g, hence further justifying the application for the intake group cut-offs used in this study. The intake cut-off at 15 g per day also aligned with the results from a global study where daily nut and seed intake below the optimal range (16–25 g/day) was associated with higher all-cause mortality [30].

### Metabolic syndrome and the component conditions

Metabolic syndrome was ascertained using the criteria published by Alberti et al*.* [1]. This set of criteria was chosen because it is the most updated criteria for defining metabolic syndrome. Participants meeting the criteria for any three of the following conditions were defined as having metabolic syndrome: high fasting plasma glucose (FPG) (≥ 100 mg/dL or using insulin or taking medication for hyperglycemia), high triglycerides (≥ 150 mg/dL or taking medication for dyslipidemia), central obesity (waist circumference ≥ 102 cm in men and ≥ 88 cm in women), high blood pressure (systolic pressure ≥ 130 mmHg, diastolic pressure ≥ 85 mmHg, or taking medication for high blood pressure), and low HDL-cholesterol (< 40 mg/dL in men or < 50 mg/dL for women, or taking medication for dyslipidemia). Fasting blood samples were collected from participants and analyzed using methods detailed elsewhere [31], while medication use was self-reported by the participants. The data for FPG and triglycerides were adjusted using regression equations provided in the NHANES data documentation of the 2015–2016 and 2017–2018 cycles to account for variations in the analysis method [24].

### Demographic factors and covariates

Covariates considered in this analysis included age, sex, ethnicity (Mexican American, Other Hispanic, Non-Hispanic White, Non-Hispanic Black, and other races), family income to poverty ratio, smoking status, HEI 2015, physical activity, alcohol intake, and history of a cardiovascular event. The family income to poverty ratio was used to depict socioeconomic status and was calculated by dividing family or individual income by the poverty guidelines specific to each survey cycle and varying by family size and geographic location [24]. Smoking status was self-reported and categorized into non-smoker, former smoker, and current smoker. The HEI 2015 was used to assess adherence to the 2015–2020 Dietary Guidelines for Americans. With a range from 0 to 100, a higher score depicts a higher adherence and thus a better diet quality [32]. Physical activity was reported as a z-score based on self-reported frequency and duration of leisure-time physical activity, which were collected from participants using different questionnaires across the seven cycles of NHANES as reported in previous studies [33, 34]. The calculation of the z-score is described elsewhere [26]. Daily alcohol intake was calculated by averaging alcohol intake from the two 24-h recalls. History of a cardiovascular event, which included congestive heart failure, angina/angina pectoris, heart attack, and/or stroke, was self-reported by participants.

### Statistical analyses

This analysis utilized sampling strata, clusters, and weights in line with the NHANES analytical guidelines ensuring the results could be generalized to the US population [24]. Two-year sample weights for each NHANES cycle were combined to provide 14-year weights for the 2005–2018 survey period. The two-day dietary recall weights were used for all outcomes.

We tested for age and sex interactions in the association between the combined intake of nuts and seeds and metabolic syndrome using a multivariate model adjusted for ethnicity, family income to poverty ratio, daily alcohol intake, smoking status, HEI 2015, physical activity, and history of a cardiovascular event. While we did not find evidence of age interaction (*p* = 0.39), the sex interaction was statistically significant (*p* = 0.005), thus all subsequent analyses were conducted separately for males and females. Since approximately 50% of participants reported zero consumption of nuts and seeds, we adopted a two-part modeling approach to assess the association between nut and/or seed intake (intake of nuts alone, seeds alone, and the combined intake of nuts and seeds) and metabolic syndrome, its components, and the measurements used to define metabolic syndrome. First, we assess if consuming any amount of nuts and/or seeds was associated with differences in outcomes compared with not consuming any. Second, we investigate if the outcomes changed with the amount of nut and/or seed consumed among consumers.

For the first part of the approach, we modeled the associations using nut and/or seed intake as binary exposure variables (0 g/day vs > 0 g/day), with non-consumers being the reference group. Logistic regression analyses (odds ratios with 95% CI) were used for binary outcomes (metabolic syndrome, high fasting glucose, high triglycerides, central obesity, high blood pressure, and low HDL-cholesterol) and covariates included age, family income to poverty ratio, ethnicity (Mexican American, Non-Hispanic White, Non-Hispanic Black, Other Hispanic, and other races), smoking status (non-smoker, former smoker, current smoker), daily alcohol intake, HEI 2015, experienced a cardiovascular event (yes/no), and physical activity *z*-scores. Linear regression analyses (beta coefficients with 95% CI) were used for continuous outcomes (FPG, triglycerides, waist circumference, systolic blood pressure, and HDL-cholesterol) with the same covariates as used in logistic regression, but with the addition of medications used for managing levels of corresponding biomarkers (e.g. use of insulin and medication for hyperglycemia was adjusted for when the outcome was fasting glucose). When we modeled the associations for nut or seed intake alone, both variables were included in the same model to account for intakes of one another.

For the second part of the approach, continuous outcomes and binary outcomes were analyzed differently. For continuous outcomes, linear regression models were used to assess the linear associations between nut and/or seed intake and all continuous outcomes among consumers only, including the same set of covariates as those in the first part. Results were presented as estimated changes in unit of measurement per one-gram additional consumption of nuts and seeds. We further investigated the presence of non-linear associations between nut and/or seed intake (among consumers only) and the continuous outcomes using linear regression models with restricted cubic splines for the exposure variables, with five knots placed at the 5th, 27.5th, 50th, 72.5th, and 95th percentiles, adjusting for the same set of covariates as in the linear association models. Model fit of linear and non-linear models of each exposure and outcome pair were then compared using likelihood-ratio tests. Whenever a non-linear model showed a better fit over the linear model (likelihood-ratio test *p* < 0.05), the non-linear associations were also reported. For binary variables, logistic regression models were used to assess the associations between categories of combined intake of nuts and seeds (0 g/day, 0.1–15.0 g/day, 15.1–29.9 g/day, 30.0 g/day or above) and all binary outcomes with 0 g/day as the reference group and the same set of covariates used in the first part. The presence of linear trends across categories of combined intake of nuts and seeds was assessed using logistic regressions with the median value of each category as the independent variable and the same set of covariates was included.

All analyses were performed in R (version 4.1.1) [35] using packages “survey” [36] and “rms” [37] while nonlinear plots were produced using the “effects” package [38]. Statistical significance was set at two-sided *p* < 0.05.

## Results

### Participant characteristics

**Fig. 1:**
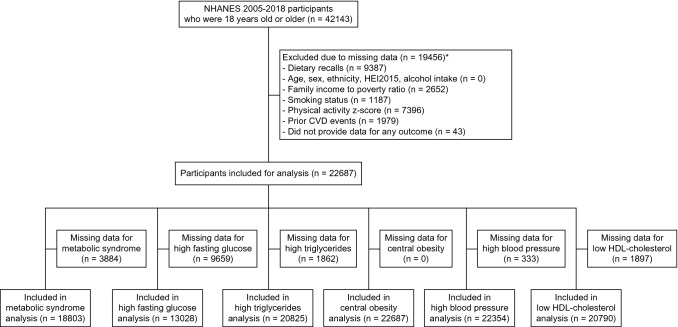
Participant exclusion flowchart for all outcomes. HDL, high-density-lipoprotein. NHANES, National Health and Nutrition Examination Survey. *Numbers of individual variables do not add up as participants could have missing data in more than one variable

A total of 22,687 participants were included in the main analysis. The inclusion flowchart is shown in Fig. 1 and the demographic characteristics are shown in Table 1. The proportion of participants who were habitual consumers of either nuts or seeds was greater in females than in males, while male consumers had a higher median intake of nuts and seeds combined than female consumers. Similar results were observed when considering nut intake alone. Around 20% females and 17% males reported to be habitual seed consumers and the median daily intake among consumers was 4 g/day in both sexes. Females were more likely to be non-smokers while males were more likely to be former smokers. Females also consumed less alcohol, had a better diet based on the HEI 2015, had lower physical activity levels, and were less likely to have a history of a cardiovascular event when compared to males. The prevalence of metabolic syndrome was approximately 50% and was similar between sexes. Males were more likely to have high fasting glucose and high triglycerides while females were more likely to have central obesity.

**Table 1:** Demographic characteristics of included participants

Characteristics	All participants	Female	Male
N	22,687	12,277	10,410
Age, years	47.3 (46.8, 47.9)	47.7 (47.2, 48.3)	46.9 (46.3, 47.5)
Combined intake of nuts and seeds			
Habitual consumer (%)^a^	60.3	62.6	57.8
Intake among consumers, g/day^b^	14.9 [14.4, 15.4]	13.1 [12.7, 13.6]	17.1 [16.4, 17.9]
Nut intake, g/day			
Habitual consumer (%)^a^	55.6	57.3	53.7
Intake among consumers, g/day^b^	14.8 [14.4, 15.3]	12.9 [12.5, 13.4]	17.2 [16.5, 17.9]
Seed intake, g/day			
Habitual consumer (%)^a^	18.5	20.3	16.5
Intake among consumers, g/day^b^	4.0 [3.6, 4.3]	3.9 [3.5, 4.3]	4.0 [3.3, 4.8]
Ethnicity, %			
Mexican American	8.7	8.5	9.0
Other hispanic	5.3	5.4	5.3
Non-hispanic white	67.8	67.3	68.3
Non-hispanic black	10.6	11.4	9.6
Other races	7.6	7.4	7.8
Family income to poverty ratio	3.0 (2.9, 3.1)	2.9 (2.8, 3.0)	3.1 (3.0, 3.2)
Smoking status, %			
Non-smoker	58.8	63.9	52.8
Former smoker	24.0	19.9	28.9
Current smoker	17.2	16.2	18.4
Alcohol intake, g/day	2.8 (2.7, 3.0)	1.8 (1.6, 1.9)	4.0 (3.8, 4.3)
HEI 2015	53.4 (53.0, 53.9)	54.8 (54.2, 55.3)	51.8 (51.3, 52.3)
Physical activity, *z*-score	0.07 (0.04, 0.09)	− 0.04 (− 0.07, − 0.01)	0.19 (0.15, 0.23)
History of cardiovascular event, %^c^	8.6	7.6	9.8
Metabolic syndrome, %^d^	50.6	50.4	51.0
High fasting glucose, %	63.0	55.7	71.2
High triglycerides, %	49.7	45.1	55.1
Central obesity, %	58.5	67.6	47.8
High blood pressure, %	39.8	38.0	41.8
Low HDL-cholesterol, %	46.2	46.0	46.4

All values are either weighted mean (95% CI) or weighted percentages, unless otherwise specified. HEI 2015, healthy eating index 2015. HDL, high-density lipoprotein

^a^Habitual consumers are those with intake > 0 g/day

^b^Values are median [interquartile range]

^c^A cardiovascular event included congestive heart failure, angina pectoris, heart attack, or stroke

^d^The definitions of each condition are as follow: metabolic syndrome: having any 3 of the following 5 conditions; high fasting glucose: ≥ 100 mg/dL or using insulin or taking medication for hyperglycemia; high triglycerides: ≥ 150 mg/dL or taking medication for dyslipidemia; central obesity: ≥ 102 cm for men and ≥ 88 cm for women; high blood pressure: systolic pressure ≥ 130 mmHg, diastolic pressure ≥ 85 mmHg, or taking medication for high blood pressure; low HDL-cholesterol: < 40 mg/dL for men or < 50 mg/dL for women, or taking medication for dyslipidemia

### Associations with metabolic syndrome and its components between consumers vs. non-consumers of nuts and/or seeds

To investigate if any amount of nut and/or seed consumption was associated with metabolic syndrome and its components compared to not consuming any, we assessed the associations between consumers and non-consumers of nuts and/or seeds and all outcomes and the results are shown in Table 2. In females, after adjusting for all covariates, habitual consumption of either nuts or seeds, as well as nuts alone, were both associated with lower odds of metabolic syndrome, high fasting glucose, central obesity, and low HDL-cholesterol. Seed intake alone in females was also associated with lower odds of high fasting glucose and low HDL-cholesterol. In males, no statistically significant associations were found, except that nut consumers had lower odds of high fasting glucose compared to non-consumers.

**Table 2:** Associations between consumer status (consumers vs non-consumers) of nuts and/or seeds and all outcomes in participants of NHANES 2005–2018^a^

Binary outcomes^d^	Sex	N	Either nuts or seeds		Nuts only^b^		Seeds only^c^	
			OR (95% CI)	*P* value	OR (95% CI)	*P* value	OR (95% CI)	*P* value
Metabolic syndrome	Male	8640	1.04 (0.88, 1.24)	0.63	1.01 (0.85, 1.20)	0.88	0.94 (0.75, 1.19)	0.62
	Female	10,163	0.83 (0.71, 0.97)	0.017*	0.82 (0.70, 0.96)	0.012*	0.84 (0.69, 1.04)	0.10
High fasting glucose	Male	6055	0.84 (0.68, 1.04)	0.10	0.81 (0.66, 0.99)	0.042*	0.95 (0.71, 1.26)	0.72
	Female	6973	0.77 (0.63, 0.94)	0.012*	0.80 (0.66, 0.97)	0.025*	0.78 (0.62, 0.97)	0.028*
High triglycerides	Male	9616	1.15 (0.99, 1.35)	0.075	1.09 (0.93, 1.28)	0.26	1.02 (0.85, 1.23)	0.82
	Female	11,209	0.98 (0.85, 1.13)	0.78	0.95 (0.83, 1.10)	0.51	0.91 (0.76, 1.08)	0.27
Central obesity	Male	10,410	0.95 (0.84, 1.08)	0.44	0.93 (0.82, 1.06)	0.27	0.88 (0.72, 1.07)	0.19
	Female	12,277	0.81 (0.70, 0.93)	0.004*	0.82 (0.71, 0.95)	0.008*	0.87 (0.74, 1.03)	0.10
High blood pressure	Male	10,285	1.06 (0.91, 1.22)	0.46	1.01 (0.86, 1.18)	0.95	1.12 (0.91, 1.37)	0.29
	Female	12,069	0.95 (0.81, 1.12)	0.55	0.87 (0.75, 1.02)	0.082	0.97 (0.81, 1.17)	0.77
Low HDL-cholesterol	Male	9500	0.93 (0.80, 1.09)	0.36	0.92 (0.79, 1.07)	0.27	0.94 (0.74, 1.19)	0.58
	Female	11,290	0.85 (0.75, 0.97)	0.019*	0.86 (0.76, 0.97)	0.017*	0.80 (0.67, 0.96)	0.018*

Continuous outcomes (units)			Beta (95% CI)	*P* value	Beta (95% CI)	*P* value	Beta (95% CI)	*P* value
Fasting glucose (mg/dL)^e^	Male	5103	− 0.89 (− 3.54, 1.76)	0.51	− 0.84 (− 3.36, 1.68)	0.51	− 2.18 (− 4.39, 0.04)	0.054
	Female	6003	− 1.41 (− 3.28, 0.46)	0.14	− 1.72 (− 3.54, 0.10)	0.064	− 0.24 (− 1.95, 1.47)	0.78
Triglycerides (mg/dL)^f^	Male	9058	− 2.68 (− 12.14, 6.77)	0.57	− 3.62 (− 13.53, 6.29)	0.47	6.36 (− 5.75, 18.48)	0.30
	Female	10,734	− 1.16 (− 7.30, 4.97)	0.71	− 5.43 (− 11.24, 0.38)	0.066	1.75 (− 6.58, 10.08)	0.68
Waist circumference (cm)	Male	10,410	− 0.67 (− 1.47, 0.14)	0.10	− 0.78 (− 1.63, 0.06)	0.07	− 1.10 (− 2.40, 0.20)	0.095
	Female	12,277	− 1.66 (− 2.68, -0.64)	0.002*	− 1.88 (− 2.89, − 0.87)	< 0.001*	− 0.48 (− 1.68, 0.71)	0.43
Systolic blood pressure (mmHg)^g^	Male	10,240	− 0.18 (− 1.15, 0.79)	0.72	− 0.27 (− 1.24, 0.71)	0.59	− 0.17 (− 1.32, 0.97)	0.77
	Female	11,983	− 0.21 (− 1.00, 0.59)	0.60	− 0.67 (− 1.55, 0.21)	0.13	0.57 (− 0.67, 1.82)	0.36
HDL-cholesterol (mg/dL)^f^	Male	9057	0.89 (0.06, 1.72)	0.035*	1.00 (0.14, 1.86)	0.023*	− 0.04 (− 1.20, 1.11)	0.94
	Female	10,730	0.87 (− 0.03, 1.77)	0.059	1.18 (0.27, 2.10)	0.012*	0.69 (− 0.43, 1.82)	0.23

Asterisks denote results that are statistically significant (*p* < 0.05). HDL, high-density lipoprotein

^a^Odds ratios were obtained using logistic regressions while beta values were obtained using linear regressions. Exposure variables are zero vs non-zero intakes, with zero intake as the reference group. All regression models included the following covariates: age, family income to poverty ratio, ethnicity (Mexican American/Non-Hispanic White/Non-Hispanic Black/Other Hispanic/Other races), smoking status (non-smoker/former smoker/current smoker), daily alcohol intake, Healthy Eating Index 2015, experienced a cardiovascular event (yes/no), and physical activity z-scores

^b^Further adjusted for seed intake in the model

^c^Further adjusted for nut intake in the model

^d^The definitions of each condition are as follow: metabolic syndrome: having any 3 of the following 5 conditions; high fasting glucose: ≥ 100 mg/dL or using insulin or taking medication for hyperglycemia; high triglycerides: ≥ 150 mg/dL or taking medication for dyslipidemia; central obesity: ≥ 102 cm for men and ≥ 88 cm for women; high blood pressure: systolic pressure ≥ 130 mmHg, diastolic pressure ≥ 85 mmHg, or taking medication for high blood pressure; low HDL-cholesterol: < 40 mg/dL for men or < 50 mg/dL for women, or taking medication for dyslipidemia

^e^Further adjusted for use of insulin or medication for hyperglycemia (yes/no)

^f^Further adjusted for use of medication for dyslipidemia (yes/no)

^g^Further adjusted for use of medication for high blood pressure (yes/no)

Regarding clinical measurements used to define metabolic syndrome, habitual consumption of either nuts or seeds was inversely associated with waist circumference in females and positively associated with HDL-cholesterol in males compared to non-consumers. Habitual nut consumption alone was inversely associated with waist circumference in females and higher HDL-cholesterol in both sexes. No statistically significant associations were observed between habitual seed intake and any of the clinical measurements in both sexes.

### Associations with measurements used to define metabolic syndrome among nut and/or seed consumers only

To explore if the measurements used to define metabolic syndrome changed with the amount of nut and/or seed consumed, we modeled the association between habitual nut and/or seed consumption with FPG, triglycerides, waist circumference, systolic blood pressure, and HDL-cholesterol among consumers only and the results are shown in Table 3. Per-gram increase in the combined intake of nuts and seeds among consumers was positively associated with waist circumference in females but not males and no statistically significant associations were observed for other outcomes. Per-gram increase in nut intake alone was positively associated with waist circumference in both males and females; whereas a per-gram increase in seed intake alone was positively associated with triglycerides and waist circumference in females, and negatively associated with systolic blood pressure in males.

**Table 3:** Associations between nut and seed intake, both combined and separately, and measurements used to define metabolic syndrome in participants of NHANES 2005–2018 who were nut and seed consumers^a^

Outcome	Sex	Nut and seed intake combined				Nut intake^b^				Seed intake^c^			
		N	Beta (95% CI)	*P* value	P _non-linear_^d^	N	Beta (95% CI)	*P* value	P _non-linear_	N	Beta (95% CI)	*P* value	P _non-linear_
Fasting glucose^e^	Male	2721	0.04 (− 0.03, 0.12)	0.26	0.13	2514	0.06 (− 0.02, 0.14)	0.13	0.47	738	0.01 (− 0.24, 0.25)	0.96	0.15
	Female	3508	0.03 (− 0.06, 0.12)	0.51	0.089	3238	0.01 (− 0.08, 0.10)	0.76	0.10	1011	0.27 (− 0.05, 0.60)	0.095	0.73
Triglycerides^f^	Male	4950	− 0.08 (− 0.41, 0.24)	0.61	0.91	4598	− 0.07 (− 0.41, 0.28)	0.69	0.82	1285	− 0.45 (− 1.78, 0.89)	0.51	0.29
	Female	6342	0.11 (− 0.20, 0.41)	0.49	0.049*	5807	0.14 (− 0.21, 0.49)	0.43	0.18	1880	1.41 (0.43, 2.38)	0.005*	0.66
Waist circumference	Male	5578	0.03 (− 0.00, 0.07)	0.056	0.35	5157	0.06 (0.02, 0.09)	0.003*	0.91	1465	− 0.00 (− 0.19, 0.19)	0.99	0.36
	Female	7147	0.07 (0.02, 0.12)	0.010*	0.14	6537	0.07 (0.02, 0.13)	0.006*	0.53	2102	0.21 (0.05, 0.37)	0.010*	0.37
Systolic blood pressure^g^	Male	5490	− 0.03 (− 0.06, 0.01)	0.12	0.028*	5072	− 0.01 (− 0.05, 0.03)	0.50	0.048*	1447	− 0.14 (− 0.27, − 0.02)	0.027*	0.49
	Female	6993	0.00 (− 0.05, 0.05)	0.99	0.071	6393	0.00 (− 0.05, 0.06)	0.90	0.10	2061	0.08 (− 0.10, 0.25)	0.39	0.54
HDL-cholesterol^f^	Male	4949	− 0.01 (− 0.04, 0.02)	0.58	0.88	4597	− 0.01 (− 0.05, 0.02)	0.46	0.87	1285	− 0.04 (− 0.15, 0.07)	0.49	0.74
	Female	6340	0.00 (− 0.04, 0.05)	0.90	0.019*	5805	− 0.02 (− 0.08, 0.03)	0.39	0.20	1879	− 0.05 (− 0.19, 0.08)	0.44	0.59

Asterisks (*) denote results that were statistically significant (*p* < 0.05). HDL, high-density-lipoprotein

^a^Only nut and seed consumers i.e. participants with non-zero habitual intake of nuts and/or seeds were included in this analysis. Beta values were obtained using linear regressions. Exposure variables are nut and/or seed intake measured in gram per day. All regression models included the following covariates: age, family income to poverty ratio, ethnicity (Mexican American/Non-Hispanic White/Non-Hispanic Black/Other Hispanic/Other races), smoking status (non-smoker/former smoker/current smoker), daily alcohol intake, Healthy Eating Index 2015, experienced a cardiovascular event (yes/no), and physical activity z-scores

^b^Only included participants with non-zero nut intake. Further adjusted for seed intake in the model

^c^Only included participants with non-zero seed intake. Further adjusted for nut intake in the model

^d^Non-linear associations were assessed using restricted cubic splines of the exposure variables with knots placed at the 5th, 27.5th, 50th, 72.5th, and 95th percentiles and including the same set of covariates as in linear models. The linear and non-linear models were then compared using likelihood ratio tests and the *p*-values were reported

^e^Further adjusted for use of insulin or medication for hyperglycemia (yes/no)

^f^Further adjusted for use of medication for dyslipidemia (yes/no)

^g^Further adjusted for use of medication for high blood pressure (yes/no)

We observed evidence of non-linear associations for the following exposure and outcome pairs (*p*_non-linear_ < 0.05, Table 3): combined intake of nuts and seeds and triglycerides in females (*p* = 0.049), combined intake of nuts and seeds and systolic blood pressure in males (*p* = 0.028), nut intake alone and systolic blood pressure in males (*p* = 0.048), and combined intake of nuts and seeds and HDL-cholesterol in females (*p* = 0.019). The plot of the estimated non-linear association indicated that triglycerides decreased with combined nut and seed intake in females (Supplemental Figure 1 ), reaching the lowest point at approximately 6 g/day. Triglycerides then increased and plateaued at intake beyond 25 g/day, although the levels remained lower than the near-zero intake level. Systolic blood pressure in males increased with combined intake of nuts and seeds until 10 g/day (Supplemental Figure 2), followed by a decrease until 30 g/day and tended to go up at higher intake. This trend was also observed with nut intake alone in males (Supplemental Figure 3), although the range of fluctuation in systolic blood pressure was within 4 mmHg for both associations. Estimated HDL-cholesterol increased with combined nut and seed intake in females (Supplemental Figure 4), peaking at 6 g/day, then slightly decreased and finally plateauing at intake beyond 12 g/day at a level higher than near-zero intake levels.

### Association between categories of nut and seed intake and metabolic syndrome and its components

To investigate if the odds of metabolic syndrome and its components change with the amount of nuts and seeds consumed, we modeled the associations between pre-specified categories of combined intake of nuts and seeds and metabolic syndrome and its components. After adjusting for all covariates, female participants consuming 0.1–15.0 g nuts and seeds per day had lower odds of having metabolic syndrome, high FPG, central obesity, and low HDL cholesterol than non-consumers. At the same level of intake, males had higher odds of having high triglycerides (Table 4). No statistically significant association was observed in all higher intake categories and both sexes, except for lower odds of high blood glucose in females at the 15.1–29.9 g/day category, and higher odds of high blood pressure in males at the 30 g/day and above category. No significant linear trend across intake categories of nuts and seeds was observed for any outcomes.

**Table 4:** Associations between categories of combined intakes of nuts and seeds and metabolic syndrome and its components in participants of NHANES 2005–2018^a^

Outcomes^b^	Sex	N	Categories of combined intake of nuts and seeds							*P*_trend_^c^
			Non-consumers (0 g/day)	0.1–15.0 g/day		15.1–29.9 g/day		≥ 30.0 g/day		
			OR	OR (95% CI)	*P* value	OR (95% CI)	*P* value	OR (95% CI)	*P* value	
Metabolic syndrome	Male	8640	1 (ref)	0.99 (0.82, 1.19)	0.90	1.06 (0.84, 1.34)	0.63	1.29 (0.94, 1.77)	0.11	0.12
	Female	10,163	1 (ref)	0.84 (0.71, 0.99)	0.036*	0.82 (0.63, 1.07)	0.15	0.74 (0.49, 1.10)	0.14	0.078
High fasting glucose	Male	6055	1 (ref)	0.82 (0.65, 1.03)	0.098	0.86 (0.62, 1.20)	0.37	0.90 (0.61, 1.31)	0.57	0.58
	Female	6973	1 (ref)	0.79 (0.64, 0.98)	0.037*	0.66 (0.50, 0.89)	0.007*	0.86 (0.56, 1.33)	0.50	0.10
High triglycerides	Male	9616	1 (ref)	1.17 (1.00, 1.37)	0.048*	1.17 (0.94, 1.45)	0.16	1.05 (0.79, 1.40)	0.74	0.64
	Female	11,209	1 (ref)	0.98 (0.85, 1.14)	0.84	1.05 (0.85, 1.30)	0.64	0.76 (0.54, 1.06)	0.11	0.34
Central obesity	Male	10,410	1 (ref)	0.91 (0.80, 1.05)	0.20	0.95 (0.79, 1.15)	0.60	1.13 (0.88, 1.43)	0.34	0.38
	Female	12,277	1 (ref)	0.78 (0.67, 0.90)	0.001*	0.92 (0.76, 1.12)	0.43	0.93 (0.67, 1.29)	0.66	0.83
High blood pressure	Male	10,285	1 (ref)	1.03 (0.88, 1.21)	0.67	0.94 (0.75, 1.18)	0.60	1.37 (1.06, 1.77)	0.017*	0.065
	Female	12,069	1 (ref)	0.98 (0.82, 1.17)	0.82	0.86 (0.66, 1.12)	0.26	0.94 (0.66, 1.33)	0.72	0.34
Low HDL-cholesterol	Male	9500	1 (ref)	0.92 (0.79, 1.07)	0.27	1.00 (0.82, 1.23)	0.97	0.88 (0.66, 1.18)	0.40	0.56
	Female	11,290	1 (ref)	0.85 (0.75, 0.97)	0.018*	0.90 (0.71, 1.14)	0.39	0.73 (0.53, 1.01)	0.058	0.08

Asterisks denote results that are statistically significant (*p* < 0.05). HDL, high-density lipoprotein

^a^The following variables were included as covariates: age, family income to poverty ratio, ethnicity (Mexican American/Non-Hispanic White/Non-Hispanic Black/Other Hispanic/Other races), smoking status (non-smoker/former smoker/current smoker), daily alcohol intake, Healthy Eating Index 2015, experienced a cardiovascular event (yes/no), and physical activity z-scores

^b^The definitions of each condition are as follow: metabolic syndrome: having any 3 of the following 5 conditions; high fasting glucose: ≥ 100 mg/dL or using insulin or taking medication for hyperglycemia; high triglycerides: ≥ 150 mg/dL or taking medication for dyslipidemia; central obesity: ≥ 102 cm for men and ≥ 88 cm for women; high blood pressure: systolic pressure ≥ 130 mmHg, diastolic pressure ≥ 85 mmHg, or taking medication for high blood pressure; low HDL-cholesterol: < 40 mg/dL for men or < 50 mg/dL for women, or taking medication for dyslipidemia

^c^*P*_trend_ were obtained using logistic regressions with median values of nut and seed intake in each nut and seed intake category as exposure variables and the same covariates as in the main models

## Discussion

In this nationally representative sample of US adults, we found that nut and seed consumers had lower odds of having metabolic syndrome and some of its components (e.g. high fasting glucose, central obesity, low HDL cholesterol) than non-consumers, but these associations were seen only in females. Congruent results were observed when nut and seed intake were considered separately, although the associations were less consistent in seed intake alone. Our analyses further demonstrated that the lower odds of metabolic syndrome and its components in females were the most pronounced at the nut and seed intake level of 0.1–15.0 g/day when compared with non-consumers (Table 4). Our study also showed for the first time in an epidemiological analysis that consumption of seeds was associated with a better metabolic profile in females i.e. a lower likelihood of having high fasting glucose and low HDL-cholesterol.

The finding that habitual nut consumption in females being inversely associated with metabolic syndrome compared to non-consumers is consistent with a cohort study involving Spanish university graduates, which observed that consuming two serves of nuts per week at baseline was inversely associated with the prevalence of metabolic syndrome after six years in females but not males [39]. This sex-specific relationship between nut consumption and metabolic benefits has also been observed in previous analyses using data from European and South Korean populations of a similar age or older [40, 41]. In support of our findings, one study that analyzed data from adult NHANES participants observed significant trends of reduction in fasting glucose and waist circumference, as well as increasing HDL-cholesterol, with the combined intake of nuts and seeds in females but not males [26]. This study also found that nut and seed consumption at all levels were inversely associated with the risk of having non-alcoholic fatty liver disease (NAFLD) in females, while this association was only observed in males with moderate nut and seed intake (15.1–30.0 g/day) [26]. This difference in NAFLD risk between male and female could possibly explain our observations in this study as NAFLD was known to be a cause of metabolic syndrome [42]. Another possible explanation is a variation in the time of nut and seed ingestion during the day, as it was previously shown that the preference in number and timing of meals, which have profound influence on metabolic health [43], could be different between sexes [44]. Similarly, a recent meta-analysis of randomized controlled trials found that the timing of nut intake during the day could affect energy expenditure in human subjects [45]. Nonetheless, we acknowledge that a mechanistic explanation is yet to be offered and this difference in the association between sexes should be verified in future randomized controlled studies and longitudinal studies.

The effect estimates for nut intake alone observed in this study were similar in direction and magnitude to those of combined intake of nuts and seeds, while the findings related to seed intake were less pronounced. This suggests that nut intake was the main contributor to the findings associated with the combined intake of nuts and seeds observed in this study, which is expected given the higher consumption of nuts compared to seeds among the included participants. Despite the weaker associations observed with seed intake, we were able to observe consistencies in results between consumption of nuts alone and seeds alone, especially the inverse associations with high fasting glucose and low HDL-cholesterol. Together with the fact that nuts and seeds had comparable nutrient content [19], our results provide support, from an epidemiological perspective, to encourage intake of these two food types for better metabolic health, supporting their combination in the same food group in the dietary guidelines. The similarities in nutrient content between nuts and seeds also imply that they can be used complimentarily or as a substitution (in the case of nut allergy) in the habitual diet. The call for consuming both nuts and seeds for optimal metabolic health is further supported by evidence that dietary patterns involving higher amount of both nuts and seeds, such as the Mediterranean diet and the plant-based Dietary Portfolio [46], were associated with lower incidence of type 2 diabetes and cardiovascular diseases [47, 48]. Interestingly, nut and seed intake was associated with improvement in overall diet quality in a previous study [49] and while this is not captured by our study design, it remains to be another possible mechanism contributing to metabolic benefits. Nonetheless, it is important to note that the clinical markers between habitual seed consumers and non-consumers were not significantly different (Table 2), which could be due to the small amount of seeds consumed (mean intake: 4 g/day) among included participants, as opposed to the high doses used in previous RCTs (usually ≥ 20 g/day). Therefore, the associations of habitual seed intake at this level with metabolic health should be further assessed in future longitudinal studies and randomized controlled trials.

Although our results supported a more favorable metabolic profile in habitual nut and seed consumers compared to non-consumers, these associations did not appear to be dose-dependent. The non-linear associations between combined intake of nuts and seeds and triglycerides and HDL cholesterol in females showed that optimum benefits were achieved at an intake of approximately 6 g/day (Table 3), while the combined intake of nuts and seeds above 15 g/day had no association with metabolic syndrome (Table 4). This was in line with the results from a meta-analysis of prospective cohort studies, which found that changes in the incidence of central obesity plateaued after nut intake exceeded 5 g/day [14], while a recently published cohort study involving Iranian adults also observed an inverse association with metabolic syndrome incidence in the second quantile of nut consumption but not in higher quantiles [50]. These findings suggested that the benefits of nut and/or seed intake on metabolic syndrome could be achieved at low levels (i.e. ≤ 15 g/day). Our findings align with the recommendation of having an ounce-equivalent (i.e. 15 g) of nuts and seeds on most days of a week [10], although the current recommendation was developed based on results of food pattern modeling [51] to ensure adequate nutrient intakes across different life stages. On the other hand, the possibility that inadequate data points at higher nut and seed intake precluding associations to be detected could not be excluded. Studies conducted using data from populations with higher nut and seed intake, such as those following the Mediterranean diet, may be able to address this limitation.

The strengths of this study include the use of a large nationally representative sample and dietary data collected using a validated method [52]. Furthermore, we estimated the habitual intake of nuts and seeds, which is an episodically consumed food group, using the MSM to account for intra-individual variation. In addition, we considered nuts and seeds in different forms, including in the whole form, as an ingredient, and in butter form, to obtain a more precise estimate of nut and seed intake. As per the guidance of the American Nutrition Society, we provided effect estimates together with 95% CI for all results [53]. On the other hand, several limitations should be considered when interpreting the findings. First, the results might be affected by reverse causation and residual confounding due to the cross-sectional nature of this study. Second, with metabolic syndrome being the primary outcome of this study, multiple secondary outcomes were analyzed and the *p*-values in this study should be interpreted carefully. Third, the intake of seeds was very low among the included participants, thereby limiting the ability of this analysis in detecting significant associations. Finally, caution must be exercised when attempting to generalize the results of this study to other populations.

## Conclusion

Intake of nuts and seeds, both combined and separately, was inversely associated with metabolic syndrome and its component conditions in females, with the associations being the most pronounced at 0.1–15.0 g/day and higher intake was not associated with lower odds. Nut consumption seemed to be the main driver of these relationships, with seed consumption showing consistent associations. Future studies should investigate the longitudinal relationships between habitual nut and/or seed intake with metabolic health outcomes, as well as reasons for the sex-dependent associations found in this study.

## Supplementary Information

Below is the link to the electronic supplementary material.

Supplementary file1 (DOCX 271 KB)

## Data Availability

The data that support the findings of this study is available from the corresponding author upon reasonable request.
