# Supplementary material for: Nut and seed consumption is inversely associated with metabolic syndrome in females but not males: findings from the 2005–2018 NHANES data
Source: Eur J Nutr. 2023 Apr 28;62(6):2415–27. doi: 10.1007/s00394-023-03157-1 (PMC10421777; doi:10.1007/s00394-023-03157-1)
Supplement: Supplementary file 1 — Supplementary file1 (DOCX 271 KB) [file 394_2023_3157_MOESM1_ESM.docx]

Supplemental figure 1 Nonlinear association between combined intake of nuts and seeds and triglycerides in female participants. Solid line depicts the modeled mean triglycerides at different combined intake of nuts and seeds after adjusting for age, sex (male/female), family income to poverty ratio, ethnicity (Mexican American/Non-Hispanic White/Non-Hispanic Black/Other Hispanic/Other races), smoking status (non-smoker/former smoker/current smoker), daily alcohol intake, Healthy Eating Index 2015, experienced a cardiovascular event (yes/no), physical activity z-scores, and use of medication for dyslipidemia (yes/no). Shaded area depicts 95% CI. Lowest value of nut and seed intake was set at 0.1 g/d. Rug at the bottom of plot area depicts data points.

Supplemental figure 2 Nonlinear association between combined intake of nuts and seeds and systolic blood pressure in male participants. Solid line depicts the modeled mean systolic blood pressure at different combined intake of nuts and seeds after adjusting for age, sex (male/female), family income to poverty ratio, ethnicity (Mexican American/Non-Hispanic White/Non-Hispanic Black/Other Hispanic/Other races), smoking status (non-smoker/former smoker/current smoker), daily alcohol intake, Healthy Eating Index 2015, experienced a cardiovascular event (yes/no), physical activity z-scores, and taking medication for high blood pressure (yes/no). Shaded area depicts 95% CI. Lowest value of nut and seed intake was set at 0.1 g/d. Rug at the bottom of plot area depicts data points.

Supplemental figure 3 Nonlinear association between nut intake alone and systolic blood pressure in female participants. Solid line depicts the modeled mean systolic blood pressure at different nut intake levels after adjusting for age, sex (male/female), family income to poverty ratio, ethnicity (Mexican American/Non-Hispanic White/Non-Hispanic Black/Other Hispanic/Other races), smoking status (non-smoker/former smoker/current smoker), daily alcohol intake, Healthy Eating Index 2015, experienced a cardiovascular event (yes/no), physical activity z-scores, and use of medication for high blood pressure (yes/no). Shaded area depicts 95% CI. Lowest value of nut intake was set at 0.1 g/d. Rug at the bottom of plot area depicts data points.

Supplemental figure 4 Nonlinear association between combined intake of nuts and seeds and HDL-cholesterol in female participants. Solid line depicts the modeled mean HDL-cholesterol at different combined intake of nuts and seeds after adjusting for age, sex (male/female), family income to poverty ratio, ethnicity (Mexican American/Non-Hispanic White/Non-Hispanic Black/Other Hispanic/Other races), smoking status (non-smoker/former smoker/current smoker), daily alcohol intake, Healthy Eating Index 2015, experienced a cardiovascular event (yes/no), physical activity z-scores, and use of medication for dyslipidemia (yes/no). Shaded area depicts 95% CI. Lowest value of nut and seed intake was set at 0.1 g/d. Rug at the bottom of plot area depicts data points.
